# Supplementary material for: Does the Lightning Process Training Programme Reduce Chronic Fatigue in Adolescent and Young Adult Cancer Survivors? A Mixed-Methods Pilot Study
Source: Cancers (Basel). 2021 Aug 13;13(16):4076. doi: 10.3390/cancers13164076 (PMC8394577; doi:10.3390/cancers13164076)
Supplement: Supplementary file 1 [file cancers-13-04076-s001.zip › cancers-1323437-supplementary.pdf]

Supplementary Materials

Does the Lightning Process Training Programme Reduce Chronic Fatigue in Adolescent and Young Adult Cancer Survivors? A Mixed-Methods Pilot Study

Lena Fauske, Øyvind S. Bruland, Alv A. Dahl, Aase Myklebostad and Silje E. Reme

Table S1. The Fatigue Questionnaire: total fatigue.

Patient No.	TF Baseline	TF 3 Months	Change B → 3 *	TF 6 Months	Change B → 6 *	Change 3 → 6 **
**1**	22	9	Yes	12	Yes	No
**2**	27	7	Yes	5	Yes	No
**3**	22	9	Yes	8	Yes	No
**4**	21	5	Yes	4	Yes	No
**5**	29	7	Yes	9	Yes	No
**6**	24	15	Yes	19	Yes	Yes, worse
**7**	30	29	No	30	No	No
**8**	28	-	-	-	-	-
**9**	25	20	Yes	15	Yes	Yes, improved
**10**	19	7	Yes	17	No	Yes, improved
**11**	28	6	Yes	2	Yes	Yes, improved
**12**	20	7	Yes	5	Yes	No
**13**	12	13	Yes	-	-	-
**Mean (SD)**	23 (5)	11 (7)	$p < 0.001$	12 (8)	$p < 0.001$	$p = 0.73$

TF: total fatigue; SD: standard deviation; →: from baseline (B) to actual month; * More than 0.5 SD at baseline = 2.55; ** More than 0.5 SD at 3 months = 3.55.

Table S2. The Patient Health Questionnaire: sum score.

Patient No.	PHQ-9 at Baseline	PHQ-9 at 3 Months	Change B → 3 *	PHQ-9 at 6 Months	Change B → 6*	Change 3 → 6**
**1**	12	10	No	11	No	No
**2**	9	5	Yes	3	Yes	Yes
**3**	17	13	Yes	7	Yes	Yes
**4**	13	3	Yes	3	Yes	No
**5**	18	11	Yes	8	Yes	Yes
**6**	20	6	Yes	18	Yes	Yes
**7**	13	11	No	11	No	No
**8**	21	-	-	-	-	-
**9**	16	7	Yes	10	Yes	Yes
**10**	12	3	Yes	10	No	Yes
**11**	23	9	Yes	9	Yes	No
**12**	12	5	Yes	4	Yes	No
**13**	9	4	Yes	-	-	-
**Mean (SD)**	15 (5)	7 (3)	$p < 0.001$	9 (4)	$p = 0.001$	$p = 0.52$

PHQ: The Patient Health Questionnaire; SD: standard deviation; →: from baseline (B) to actual month; * More than 0.5 SD at baseline (B) = 2.3; ** More than 0.5 SD at 3 months = 1.7.

Table S3. The Work and Social Adjustment Scale: sum score.

Patient No.	WSAS Baseline	WSAS 3 Months	Change B → 3 *	WSAS 6 Months	Change B → 6 *	Change 3 → 6 **
**1**	27	21	Improved	24	Improved	Worse
**2**	19	15	Improved	7	Improved	Improved
**3**	24	20	Improved	17	Improved	Improved
**4**	25	5	Improved	4	Improved	Unchanged
**5**	18	15	Improved	8	Improved	Improved
**6**	23	11	Improved	13	Improved	Unchanged
**7**	37	33	Improved	35	Unchanged	Unchanged
**8**	25	-	-	-	-	-
**9**	25	22	Improved	21	Improved	Unchanged
**10**	28	25	Improved	23	Improved	Unchanged
**11**	27	15	Improved	18	Improved	Unchanged
**12**	20	9	Improved	11	Improved	Unchanged
**13**	35	26	Improved	-	-	-
**Mean** (**SD**)	25 (5)	17 (8)	$p < 0.001$	17 (9)	$p < 0.001$	$p = 0.45$

WSAS: The Work and Social Adjustment Scale; SD: standard deviation; →: from baseline (B) to actual month; * More than 0.5 SD at baseline = 2.65; ** More than 0.5 SD at 3 months = 3.95.

Table S4. The SF-36: Mental Composite Scale.

Patient No.	MCS Baseline	MCS 3 Months	Change B → 3 *	MCS 6 Months	Change B → 6 *	Change 3 → 6 **
**1**	58	50	Worse	67	Improved	Improved
**2**	37	43	Improved	45	Unchanged	Unchanged
**3**	22	34	Improved	43	Improved	Improved
**4**	38	57	Improved	52	Improved	Worse
**5**	21	Not Complete	-	-	-	-
**6**	22	51	Improved	27	Unchanged	Worse
**7**	45	52	Improved	52	Improved	Unchanged
**8**	33	-	-	-	-	-
**9**	41	57	Improved	40	Unchanged	Worse
**10**	43	51	Improved	30	Worse	Worse
**11**	19	50	Improved	44	Improved	Worse
**12**	24	48	Improved	46	Improved	Unchanged
**13**	51	53	Unchanged	-	-	-
**Mean** (**SD**)	35 (13)	50 (7)	$p = 0.003$	45 (11)	$p = 0.025$	$p = 0.29$

SF-36: Short-Form Health Survey 36; * More than 0.5 SD at baseline = 6.5; ** More than 0.5 SD at 3 months = 3.5.

Table S5. The SF-36: Physical Composite Scale.

Patient No.	PCS Baseline	PCS 3 Months	Change B → 3 *	PCS 6 Months	Change B → 6 *	Change 3 → 6 **
**1**	25	28	Unchanged	24	Unchanged	Unchanged
**2**	39	60	Improved	60	Improved	Unchanged
**3**	45	44	Unchanged	50	Improved	Improved
**4**	34	47	Improved	52	Improved	Unchanged
**5**	37	Not Complete	-	-	-	-
**6**	30	33	Unchanged	32	Unchanged	Unchanged
**7**	25	26	Unchanged	26	Unchanged	Unchanged
**8**	34	-	-	-	-	-
**9**	36	34	Unchanged	36	Unchanged	Unchanged
**10**	36	42	Improved	50	Improved	Unchanged
**11**	45	49	Unchanged	52	Improved	Unchanged
**12**	57	55	Unchanged	54	Unchanged	Unchanged
**13**	40	30	Worse	-	-	-
**Mean (SD)**	37 (9)	41 (11)	$p = 0.20$	44 (13)	$p = 0.04$	$p = 0.16$

SF-36: Short-Form Health Survey 36; SD: standard deviation; * More than 0.5 SD at baseline (B) = 4.5; ** More than 0.5 SD at 3 months = 5.5.

Table S6. Correlations between various PROMS.

	Correlation Coefficient			
Spearman's rho at baseline	PHQ-9 SUM 0	FQ Total Score		WSAS SUM 0
PHQ-9 SUM 0	1.00	0.58 *		0.17
FQ Total Score	0.58 *	1.00		0.17
WSAS SUM 0	0.17	0.17		1.00
Spearman's rho at 6 months	WSAS SUM 6	CSQ-8 SUM 6	FQ Total Score	PHQ-9 SUM 6
WSAS SUM 6	1.00	0.40	0.62 *	0.76 **
CSQ-8 SUM 6	0.40	1.00	0.72 *	0.54
FQ Total Score	0.62 *	0.72 *	1.00	0.80 **
PHQ-9 SUM 6	0.76 **	0.54	0.80 **	1.00

PHQ-9: Patient Health Questionnaire 9; FQ: Fatigue Questionnaire; WSAS: Work and Social Adjustment Scale; CSQ-8: Client Satisfaction 8; * Correlation is significant at the 0.05 level (2-tailed); ** Correlation is significant at the 0.01 level (2-tailed).

© 2021 by the authors. Licensee MDPI, Basel, Switzerland. This article is an open access article distributed under the terms and conditions of the Creative Commons Attribution (CC BY) license (http://creativecommons.org/licenses/by/4.0/).
